# Supplementary material for: miR-329– and miR-495–mediated Prr7 down-regulation is required for homeostatic synaptic depression in rat hippocampal neurons
Source: Life Sci Alliance. 2022 Sep 23;5(12):e202201520. doi: 10.26508/lsa.202201520 (PMC9510147; doi:10.26508/lsa.202201520)

Used as representative image

Fig 1C + part of D (PTX effect whole cell lysate + rep4 compartmentalized)

coomassie blue (for ladder visualization)

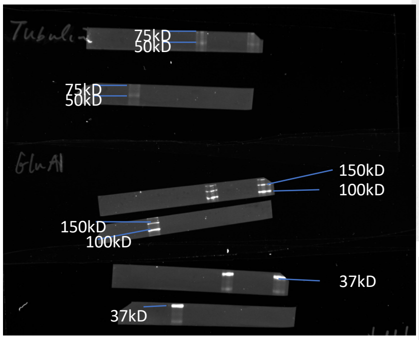

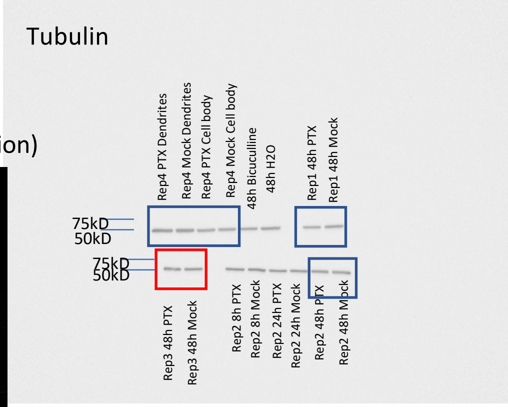

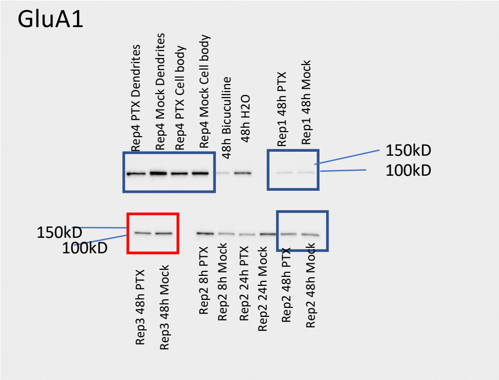

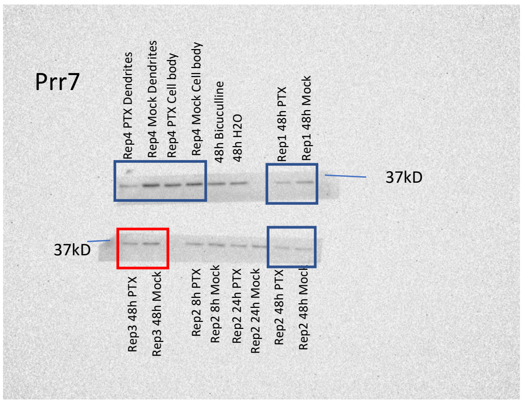

note orientation flipped to maintain EtOH -> PTX order

Fig 1D (PTX effect compartmentalized cell lysate)

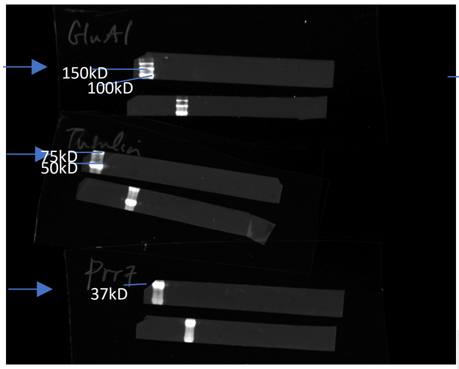

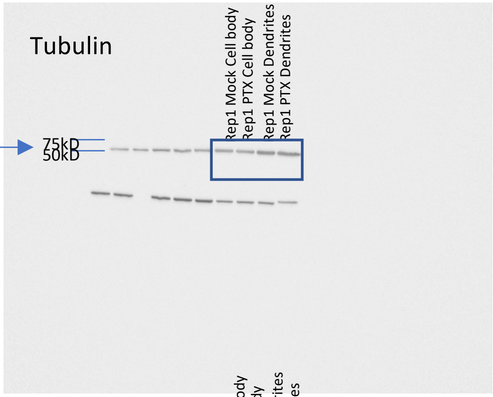

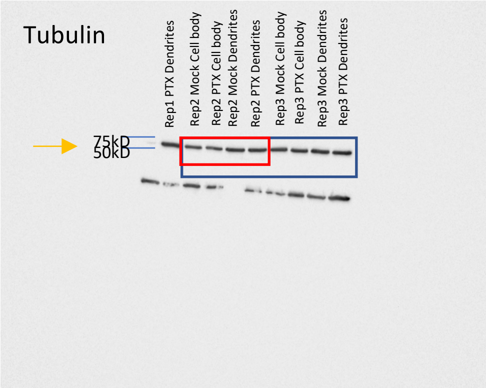

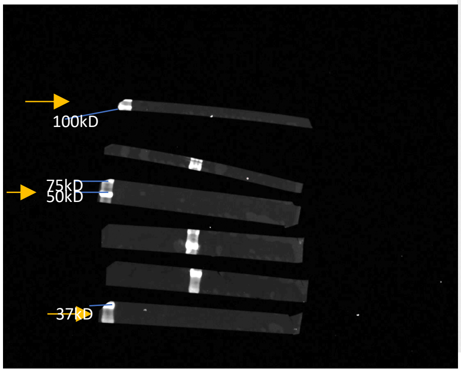

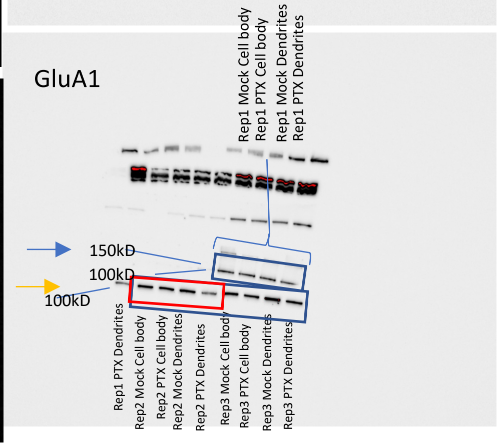

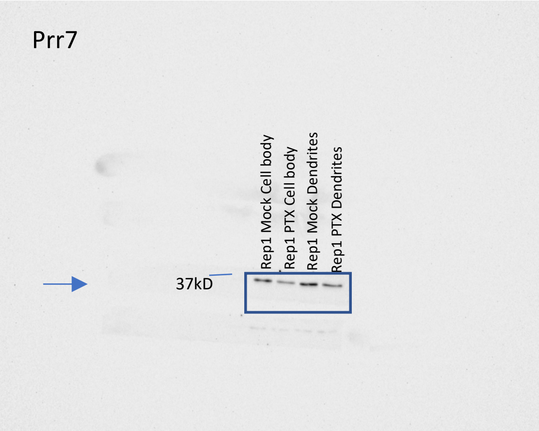

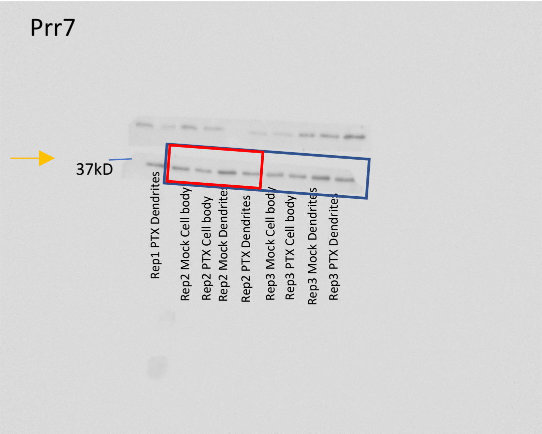

Supplement: Supplementary file 2 [file LSA-2022-01520_SdataF1.2.pdf]
