# Supplementary material for: miR-329– and miR-495–mediated Prr7 down-regulation is required for homeostatic synaptic depression in rat hippocampal neurons
Source: Life Sci Alliance. 2022 Sep 23;5(12):e202201520. doi: 10.26508/lsa.202201520 (PMC9510147; doi:10.26508/lsa.202201520)
Supplement: Supplementary file 4 [file LSA-2022-01520_SdataF2.2.pdf]

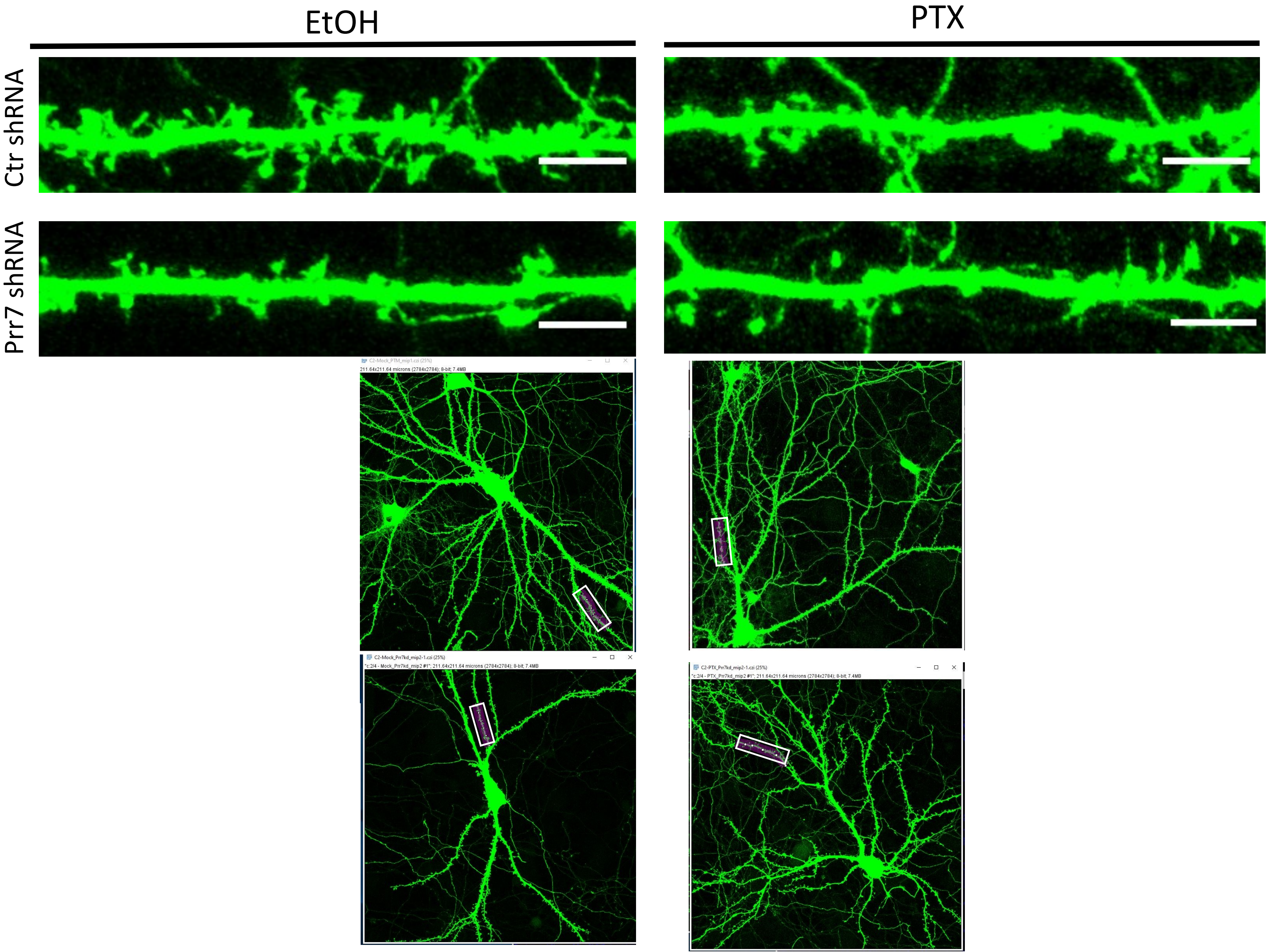
EtOH
PTX
Ctr shRNA
Prr7 shRNA

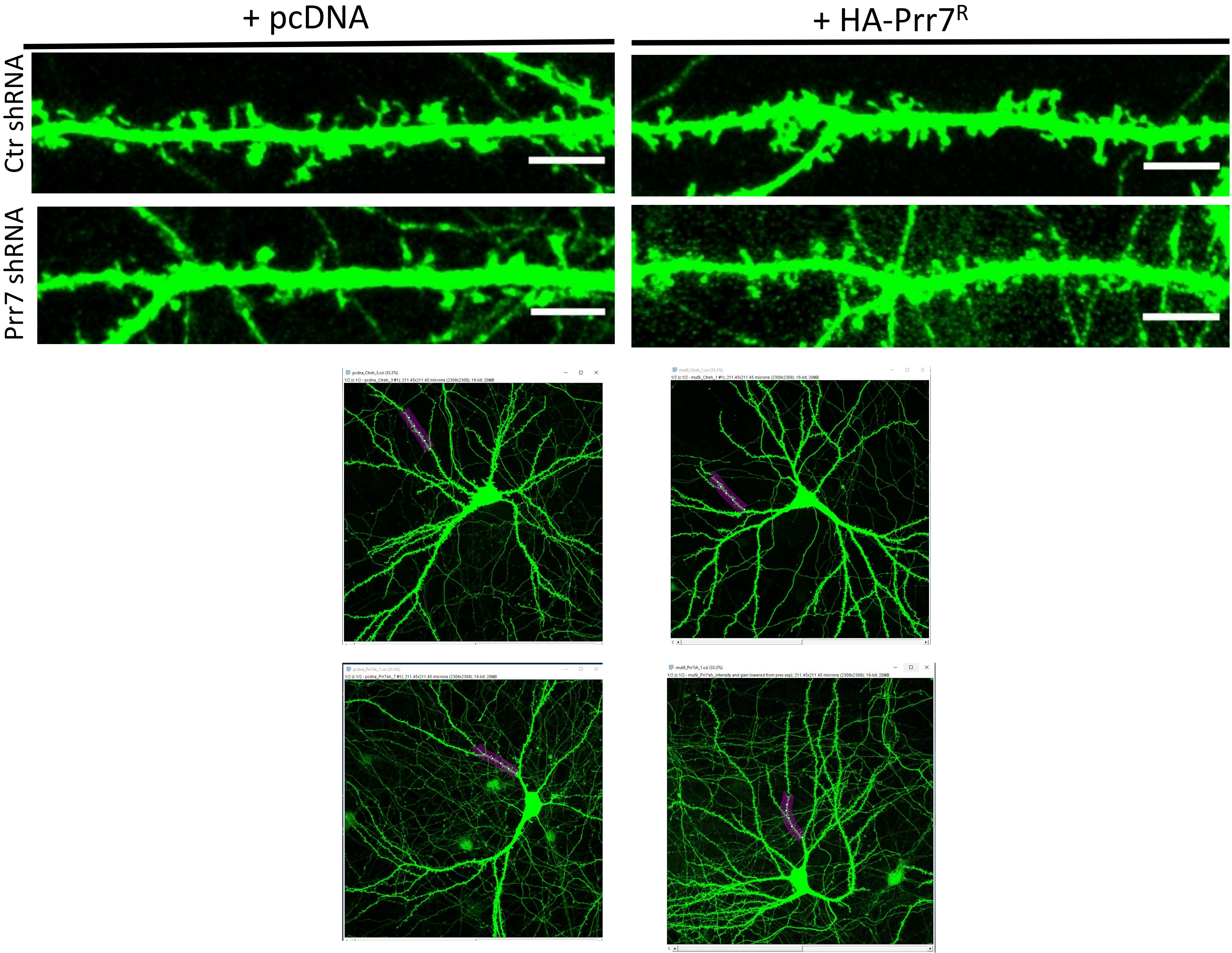
+ pcDNA
+ HA-Prr7R
Ctr shRNA
Prr7 shRNA

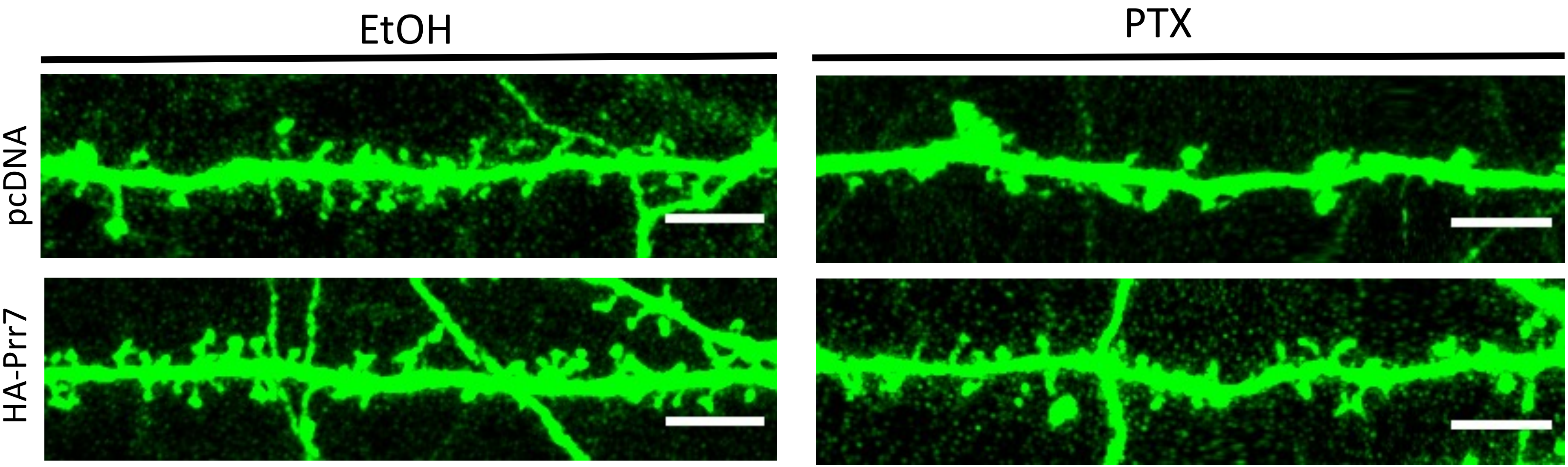
EtOH
PTX
pcDNA
HA-Prr7

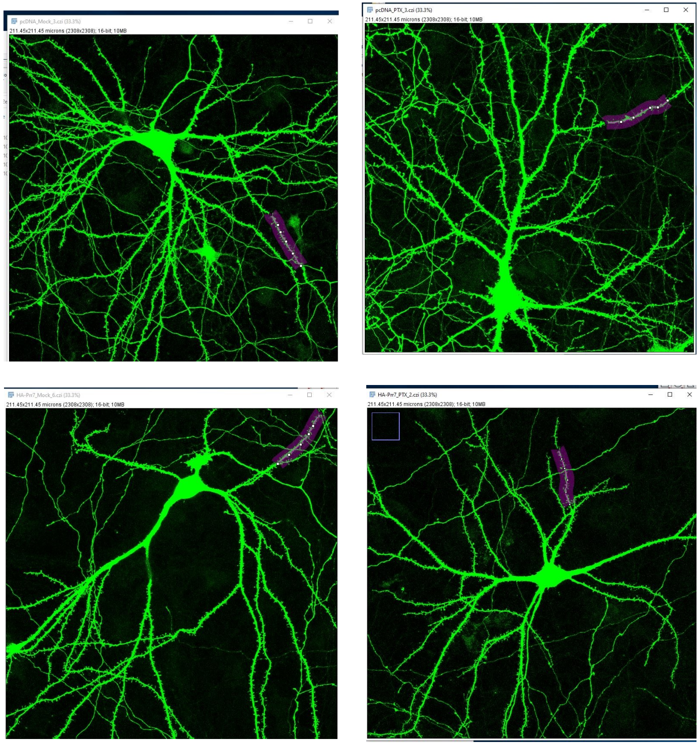
pcDNA_Mock_3.czi (33.3%)
211.45x211.45 microns (2308x2308); 16-bit; 10MB
pcDNA_PTX_3.czi (33.3%)
211.45x211.45 microns (2308x2308); 16-bit; 10MB
HA-Prr7_Mock_6.czi (33.3%)
211.45x211.45 microns (2308x2308); 16-bit; 10MB
HA-Prr7_PTX_2.czi (33.3%)
211.45x211.45 microns (2308x2308); 16-bit; 10MB

Fig 2D (GluA1 western with Prr7 shRNA)

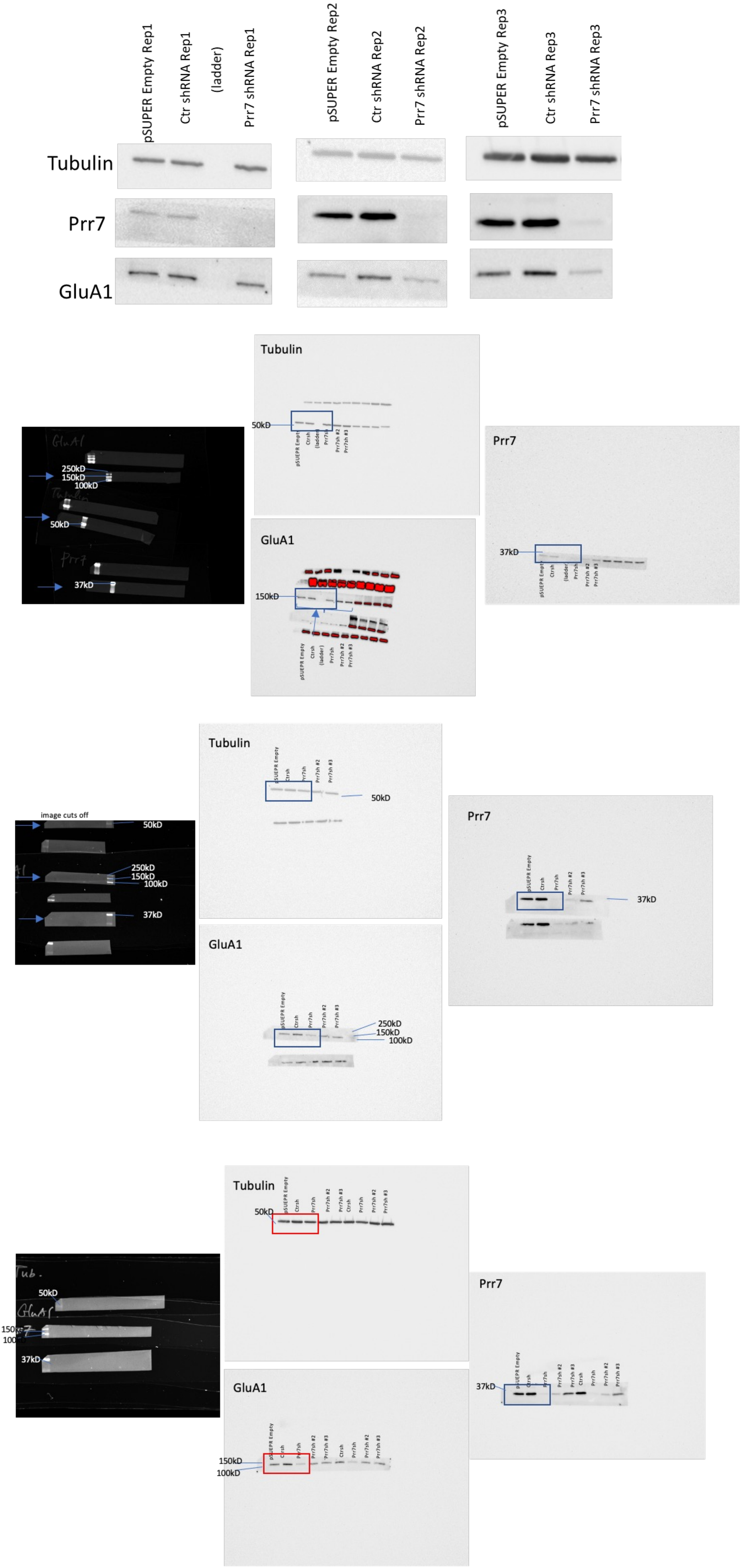

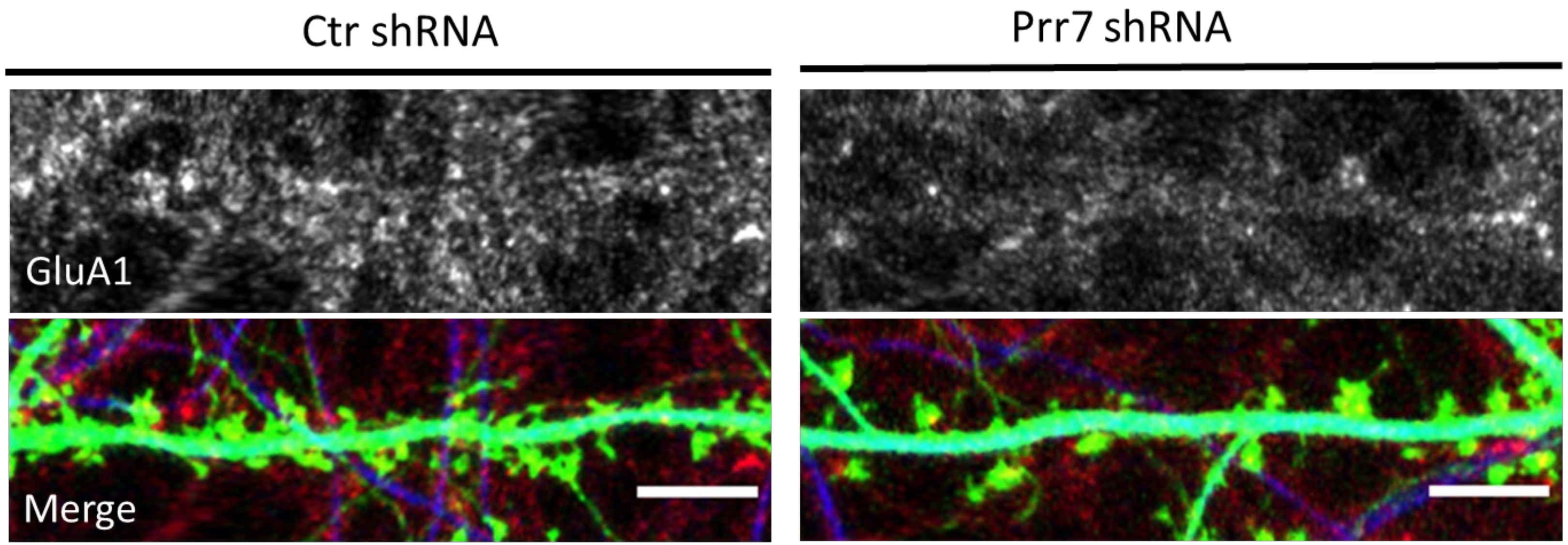
Ctr shRNA
Prr7 shRNA
GluA1
Merge

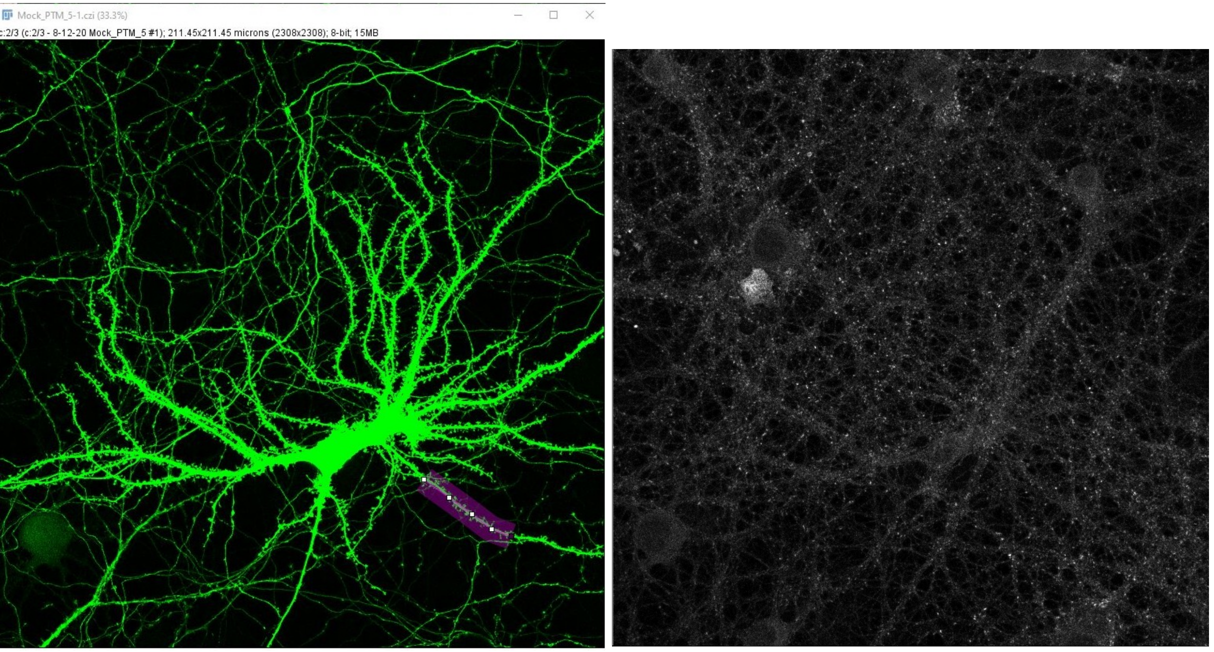
Mock_PTM_5-1.czi (33.3%)
c:2/3 (c:2/3 - 8-12-20 Mock_PTM_5 #1); 211.45x211.45 microns (2308x2308); 8-bit; 15MB

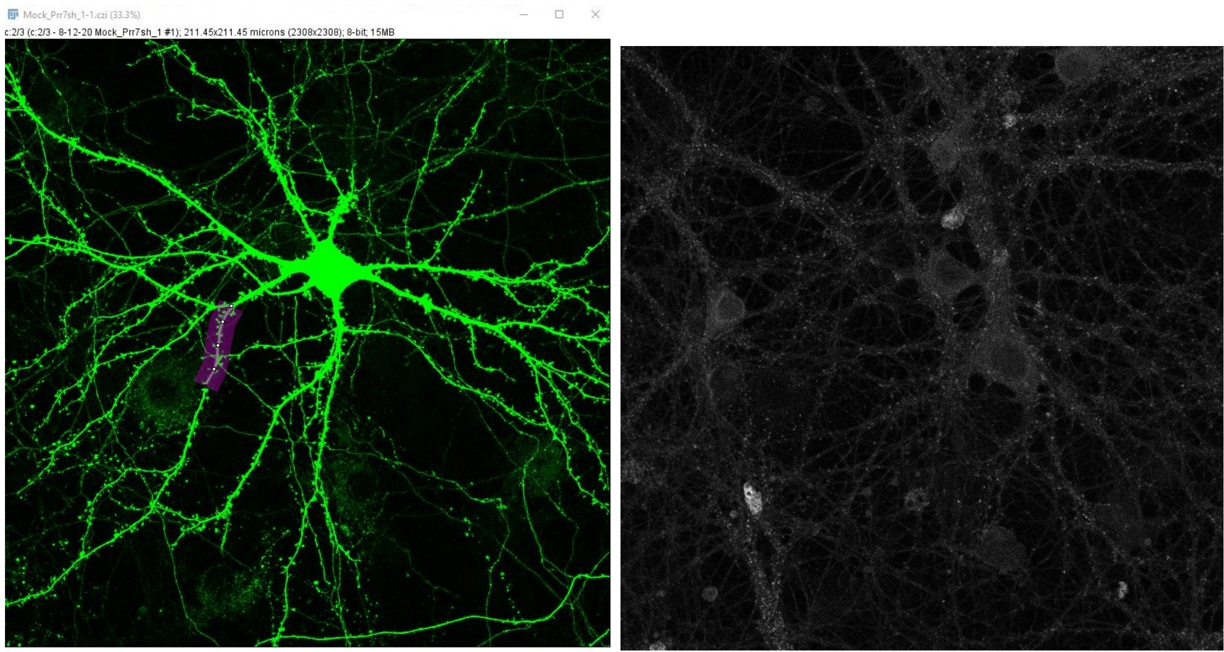
Mock_Prr7sh_1-1.czi (33.3%)
c:2/3 (c:2/3 - 8-12-20 Mock_Prr7sh_1 #1); 211.45x211.45 microns (2308x2308); 8-bit; 15MB

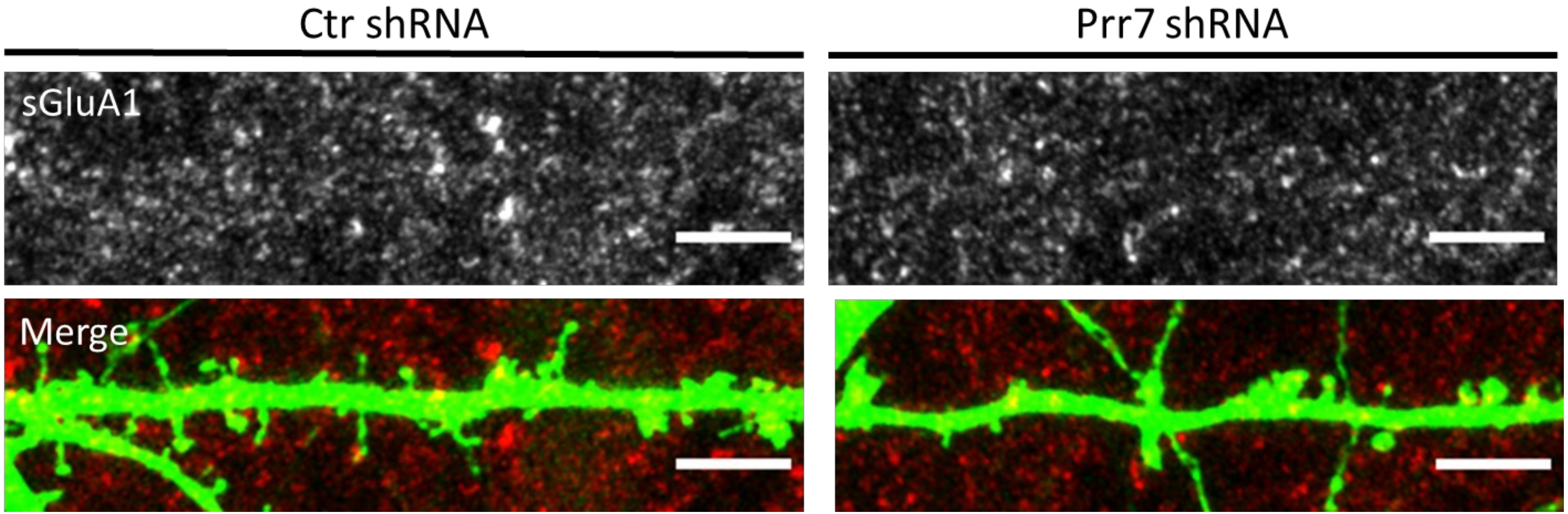
Ctr shRNA
Prr7 shRNA
sGluA1
Merge

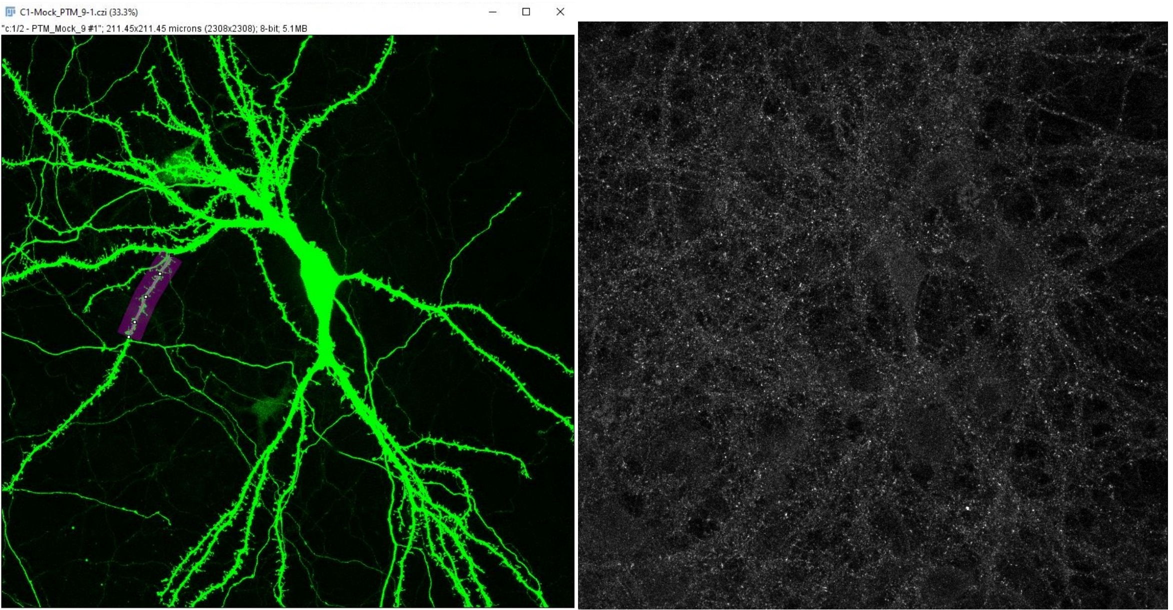
C1-Mock_PTM_9-1.czi (33.3%)
"c:1/2 - PTM_Mock_9 #1"; 211.45x211.45 microns (2308x2308); 8-bit; 5.1MB

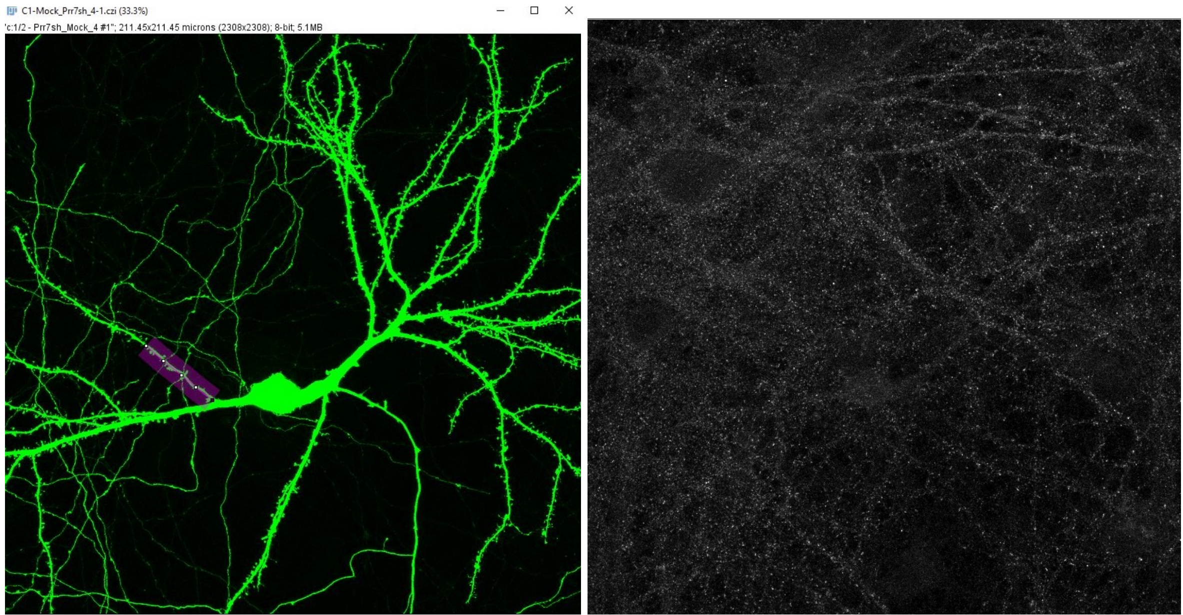
C1-Mock_Prr7sh_4-1.czi (33.3%)
'c:1/2 - Prr7sh_Mock_4 #1"; 211.45x211.45 microns (2308x2308); 8-bit; 5.1MB
