# Supplementary figures and images for: miR-329– and miR-495–mediated Prr7 down-regulation is required for homeostatic synaptic depression in rat hippocampal neurons
Source: Life Sci Alliance. 2022 Sep 23;5(12):e202201520. doi: 10.26508/lsa.202201520 (PMC9510147; doi:10.26508/lsa.202201520)

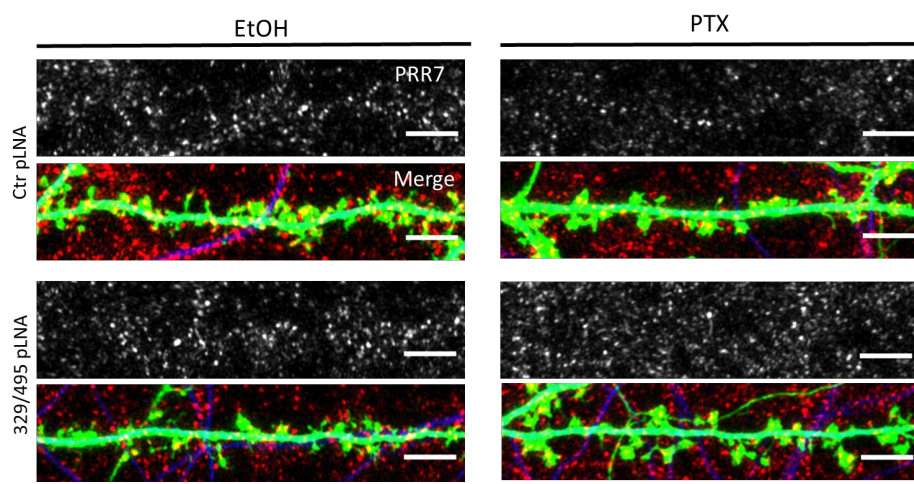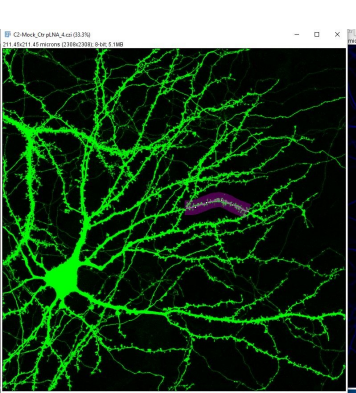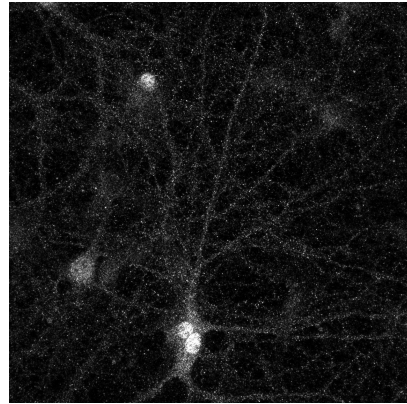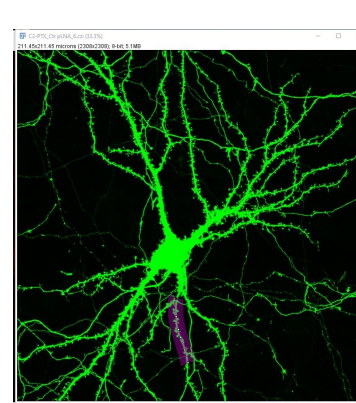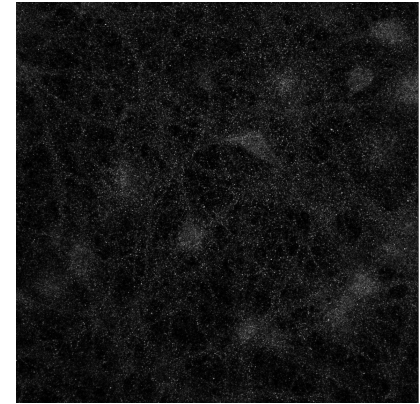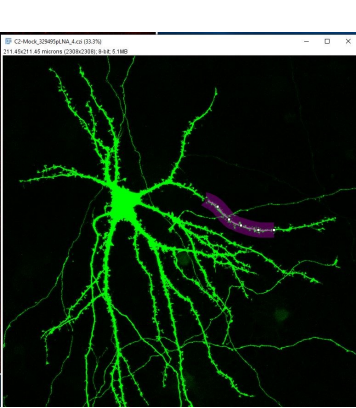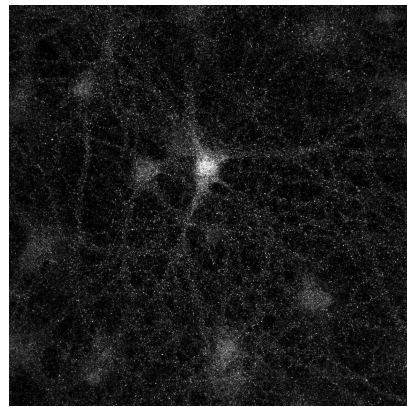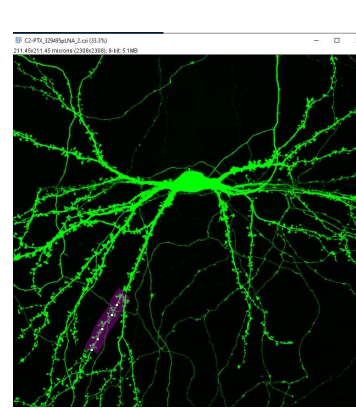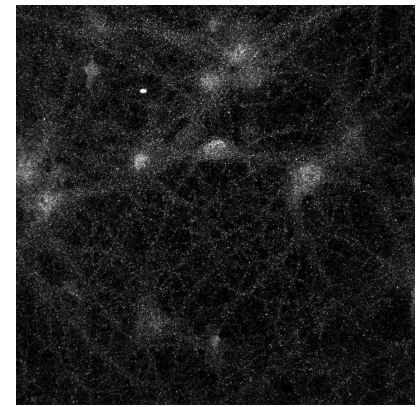

Supplement: Supplementary file 6 [file LSA-2022-01520_SdataF3.2.pdf]

Fig 6A (Leupeptin SPAR western)

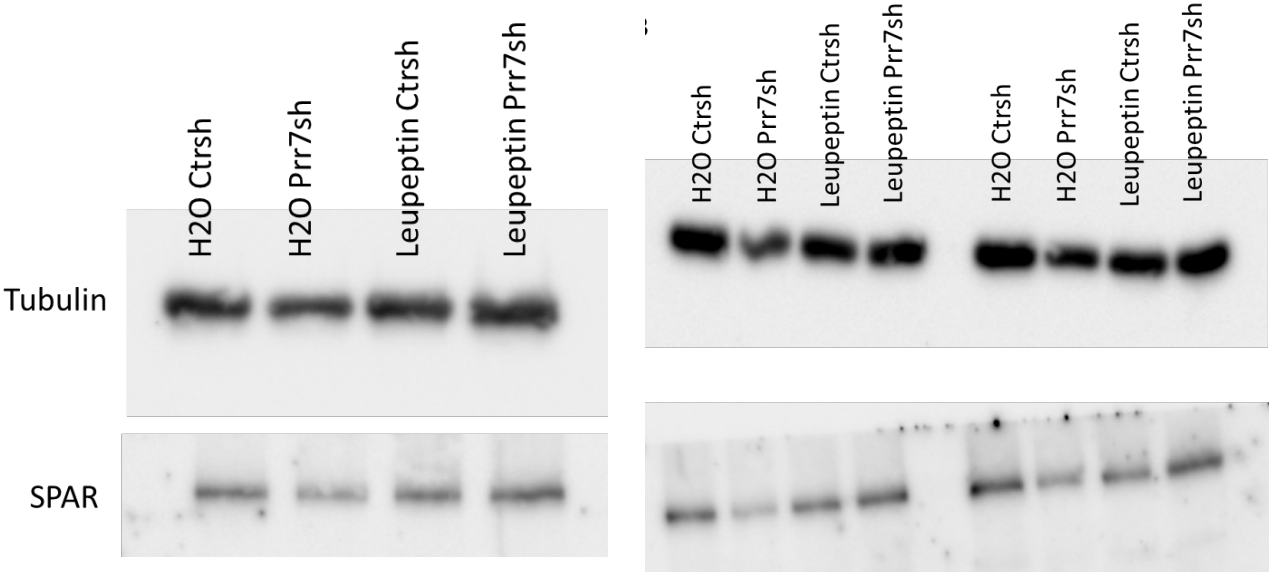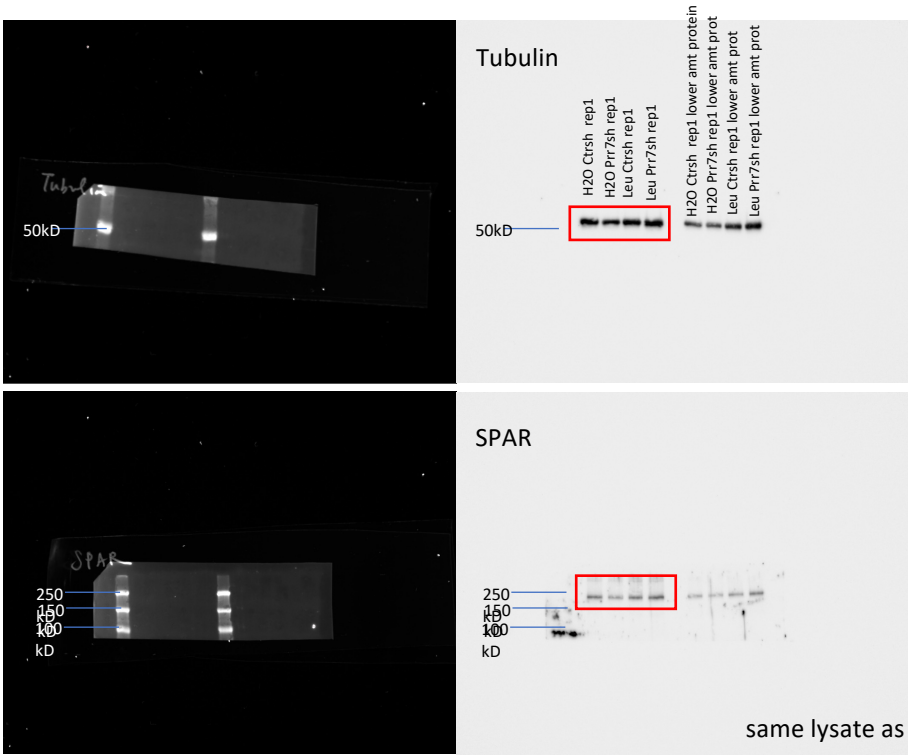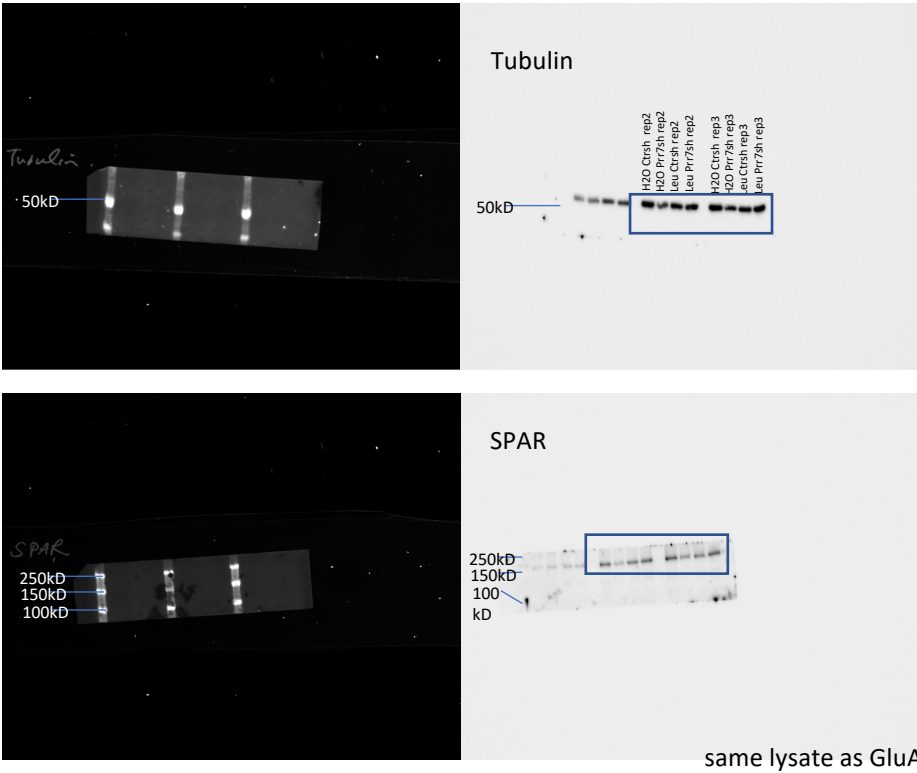

Fig 6B (Leupeptin GluA1 western)

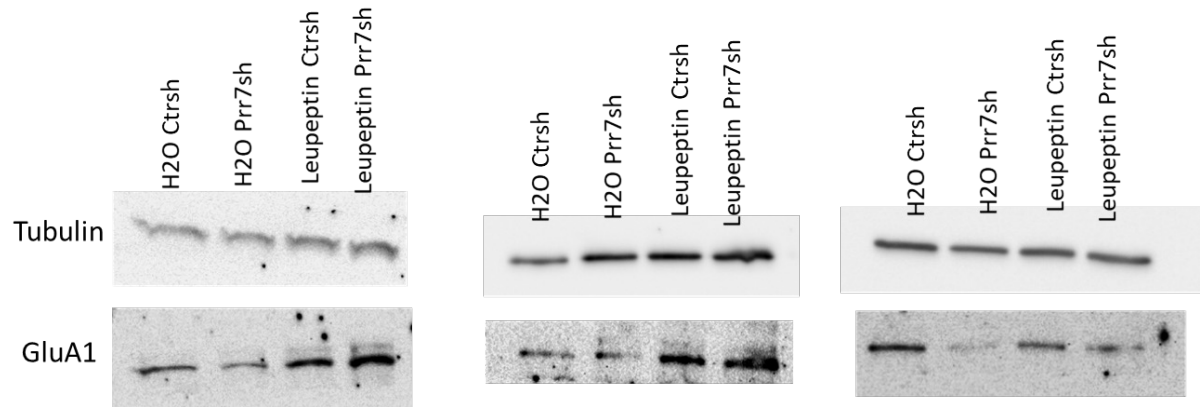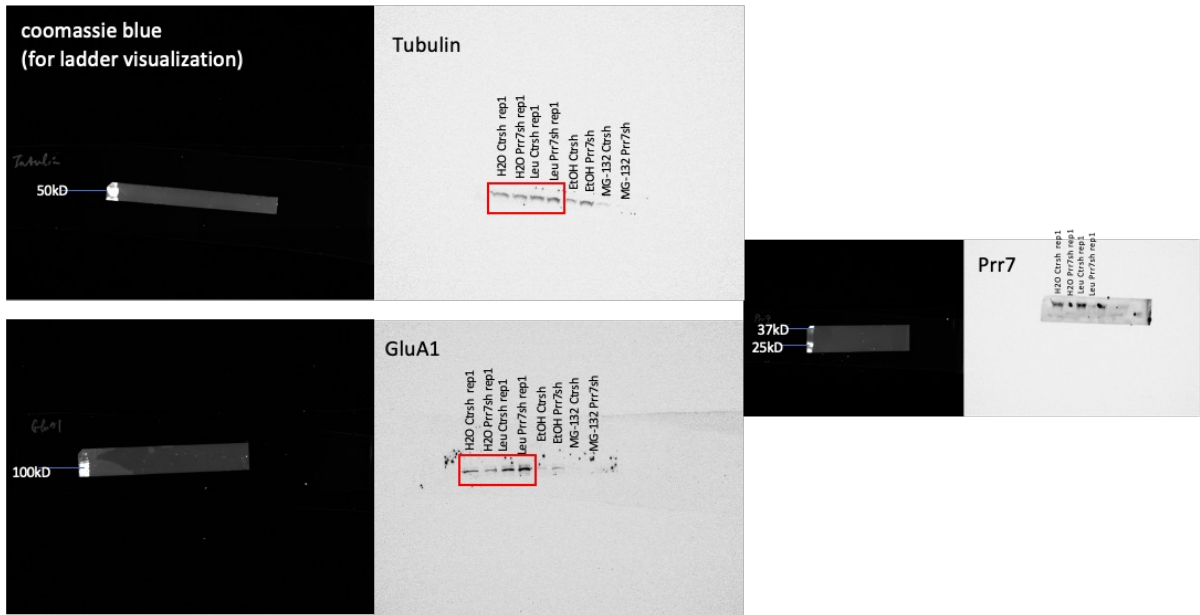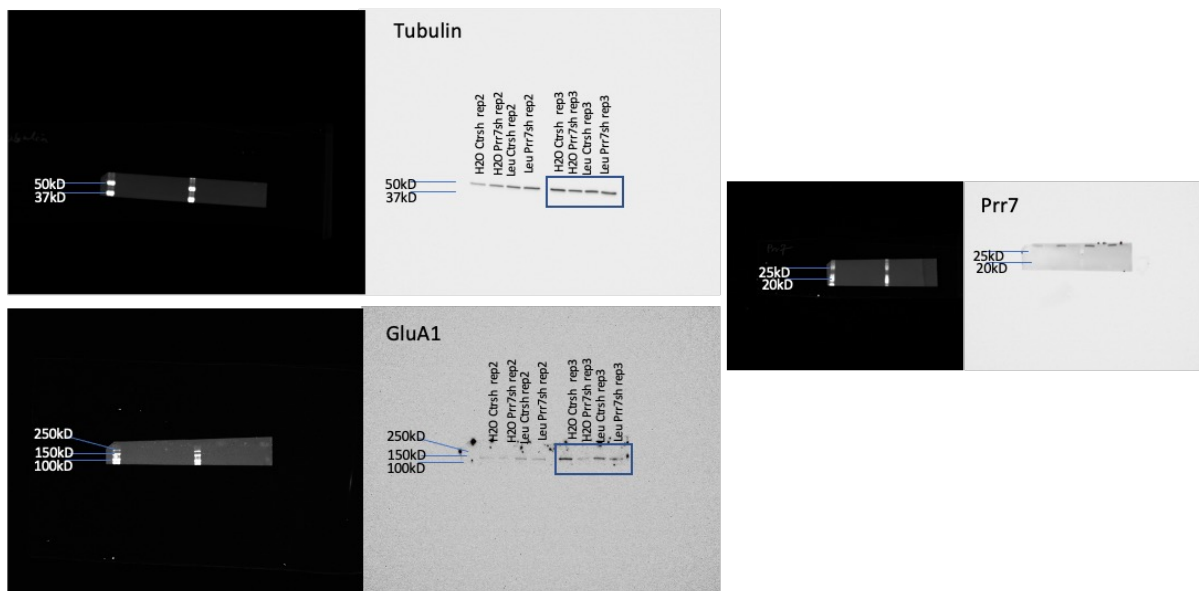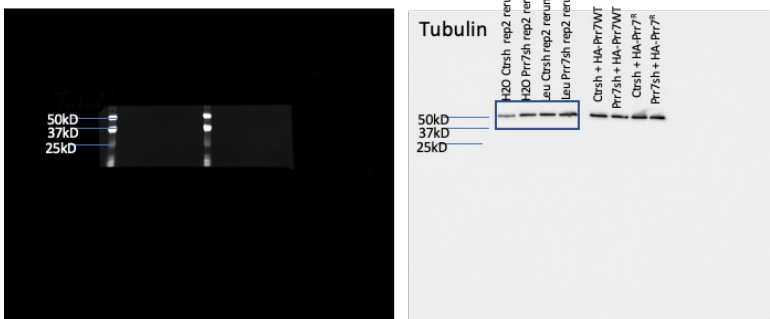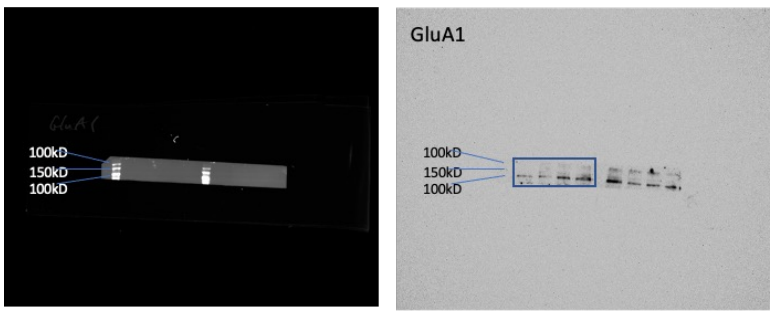

(no Prr7 probe)

Supplement: Supplementary file 12 [file LSA-2022-01520_SdataF6.2.pdf]
