# Supplementary material for: miR-329– and miR-495–mediated Prr7 down-regulation is required for homeostatic synaptic depression in rat hippocampal neurons
Source: Life Sci Alliance. 2022 Sep 23;5(12):e202201520. doi: 10.26508/lsa.202201520 (PMC9510147; doi:10.26508/lsa.202201520)

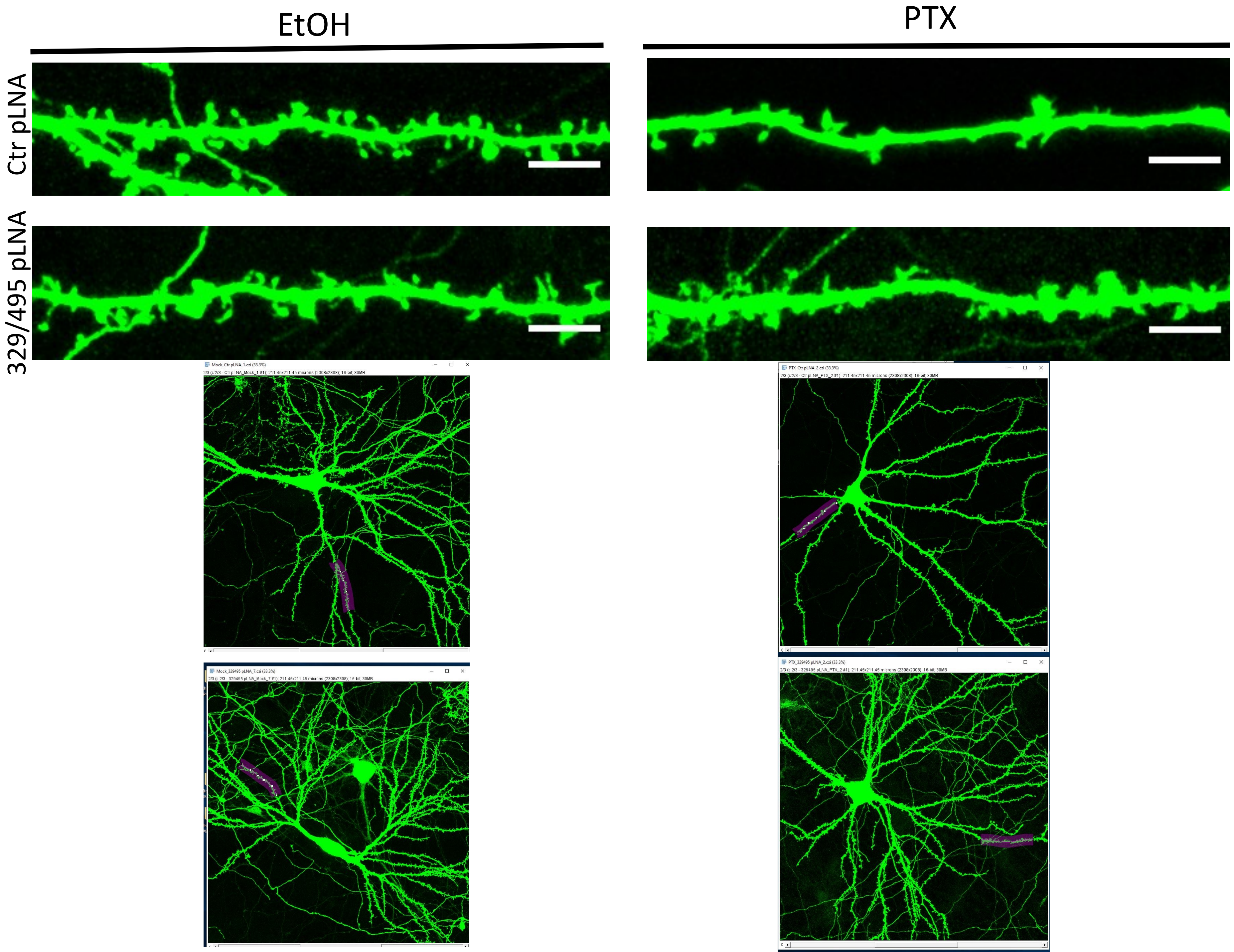
EtOH
PTX
Ctr pLNA
329/495 pLNA

PTX Ctr pLNA + Ctr shRNA

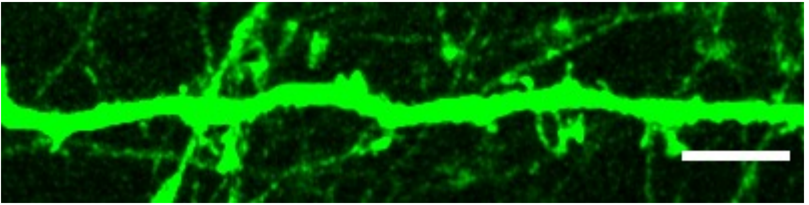

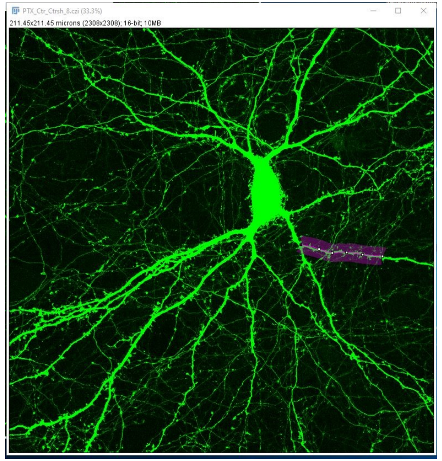

PTX 329/495 pLNA + Ctr shRNA

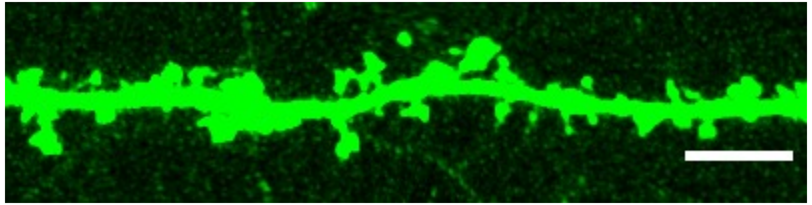

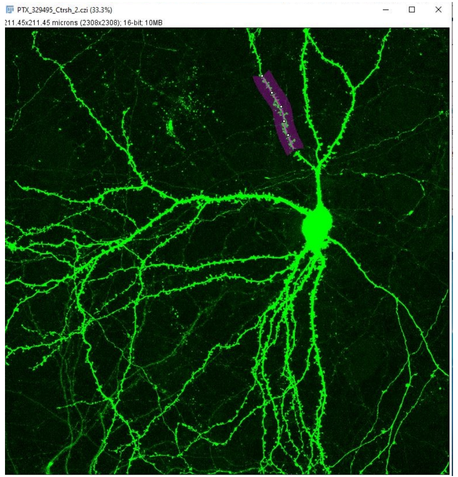

PTX 329/495 pLNA + Prr7 shRNA

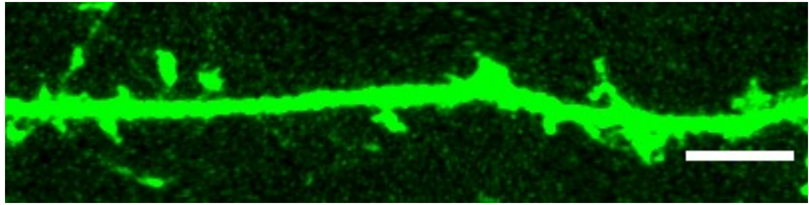

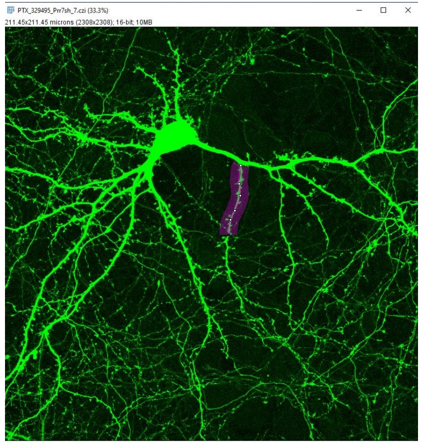

Ctr hairpin

495 hairpin

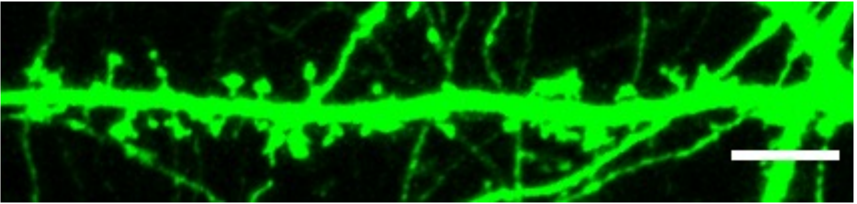

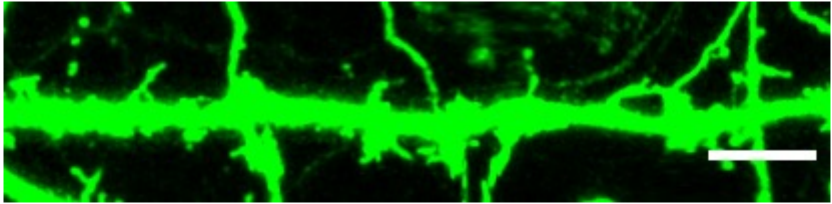

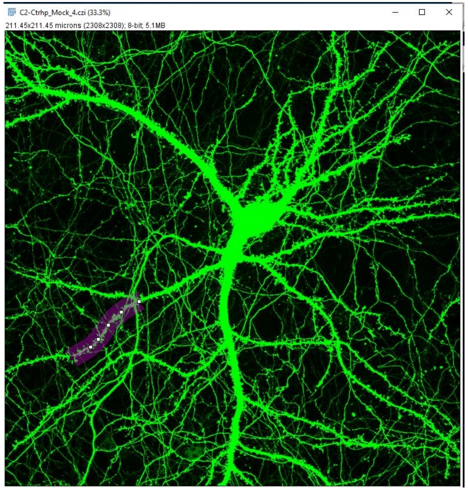

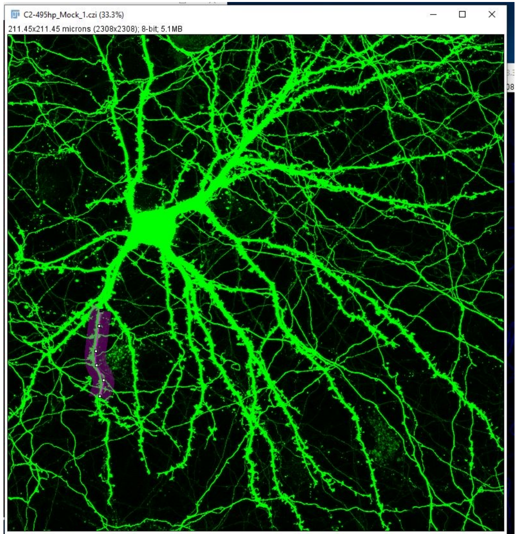

Ctr hairpin

329 hairpin

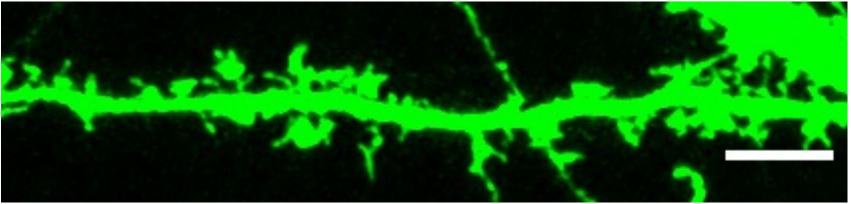

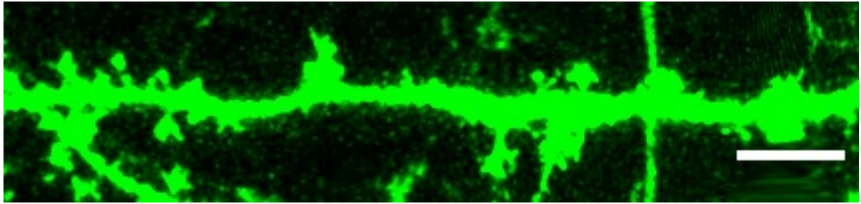

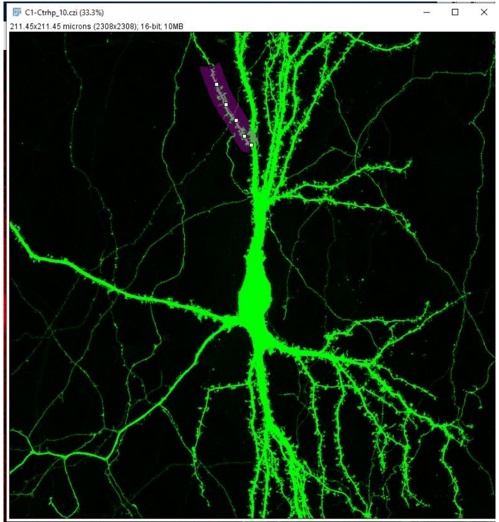

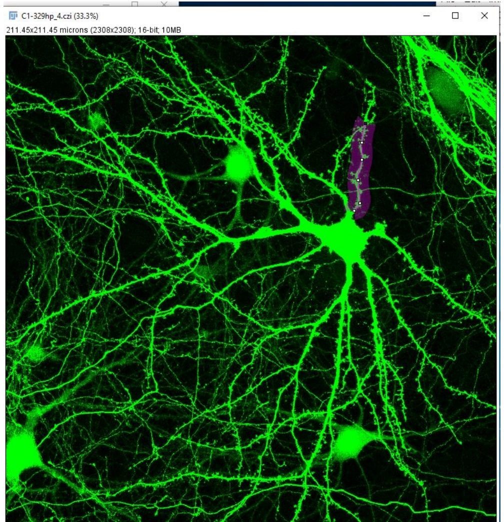

Supplement: Supplementary file 8 [file LSA-2022-01520_SdataF4.2.pdf]
