# Supplementary material for: miR-329– and miR-495–mediated Prr7 down-regulation is required for homeostatic synaptic depression in rat hippocampal neurons
Source: Life Sci Alliance. 2022 Sep 23;5(12):e202201520. doi: 10.26508/lsa.202201520 (PMC9510147; doi:10.26508/lsa.202201520)
Supplement: Supplementary file 10 [file LSA-2022-01520_SdataF5.2.pdf]

Fig 5A (SPAR western with Prr7 shRNA)

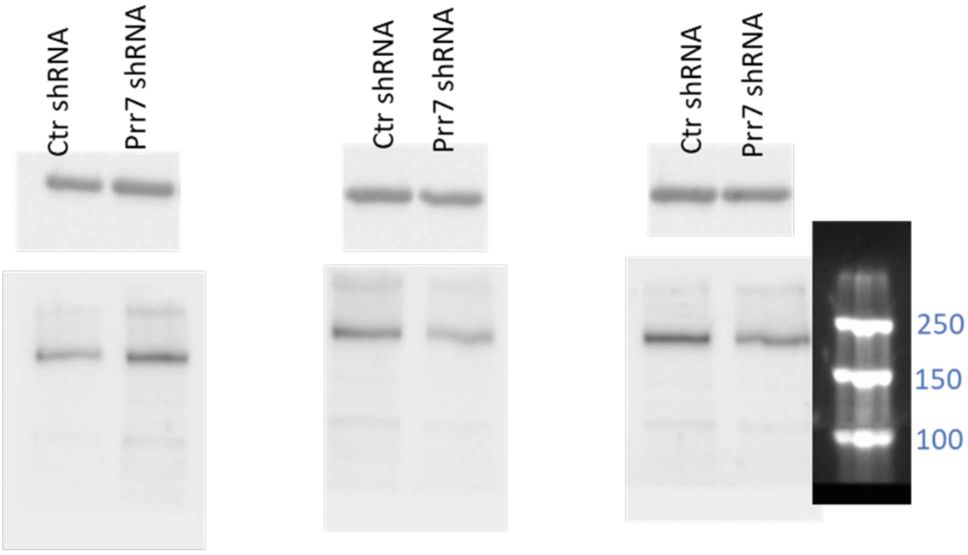

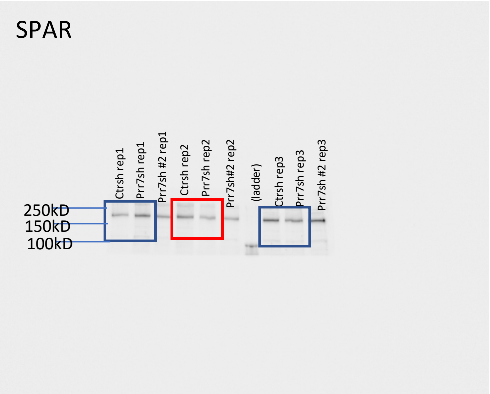

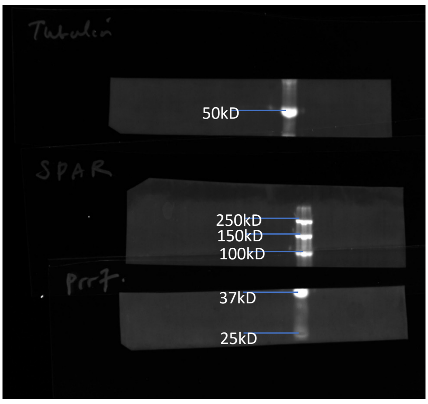

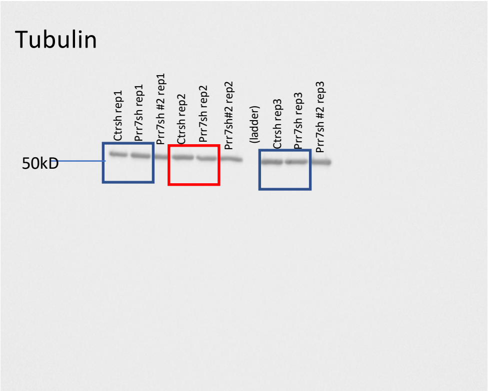

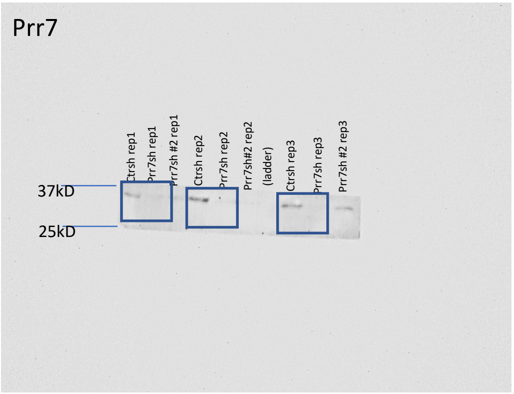

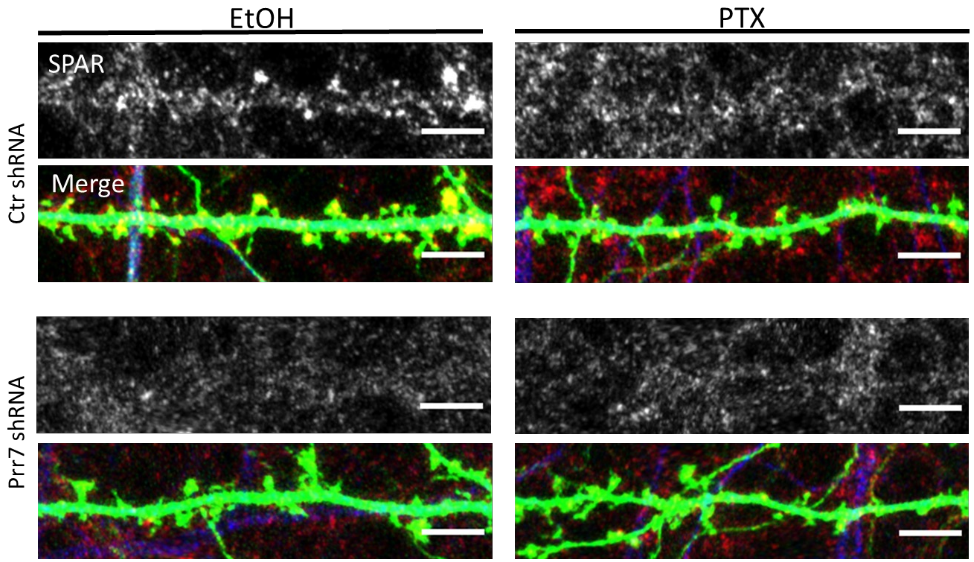
EtOH
PTX
SPAR
Merge
Ctr shRNA
Prr7 shRNA

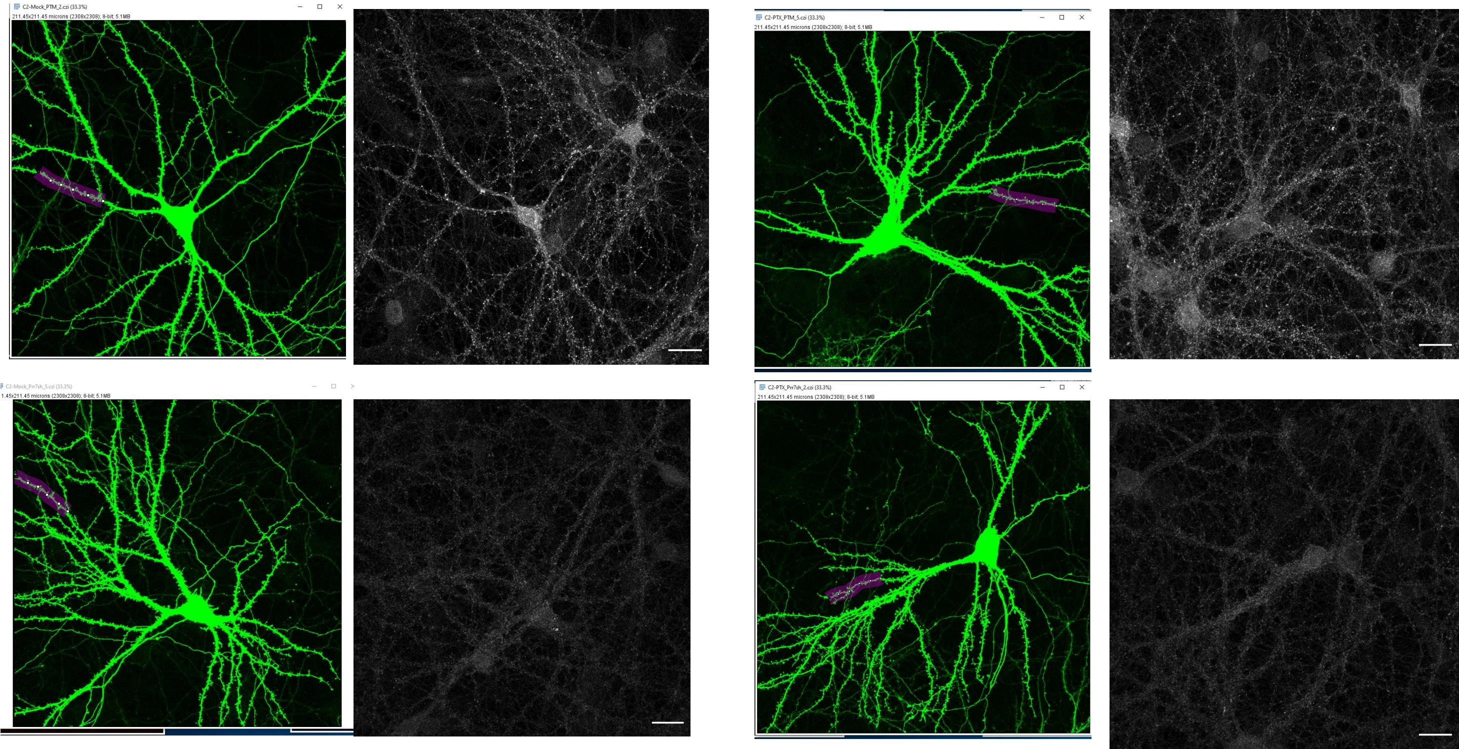
C2-Mock_PTM_2.czi (33.3%)
211.45x211.45 microns (2308x2308); 8-bit; 5.1MB
C2-PTX_PTM_5.czi (33.3%)
211.45x211.45 microns (2308x2308); 8-bit; 5.1MB
C2-Mock_Prr7sh_5.czi (33.3%)
1.45x211.45 microns (2308x2308); 8-bit; 5.1MB
C2-PTX_Prr7sh_2.czi (33.3%)
211.45x211.45 microns (2308x2308); 8-bit; 5.1MB

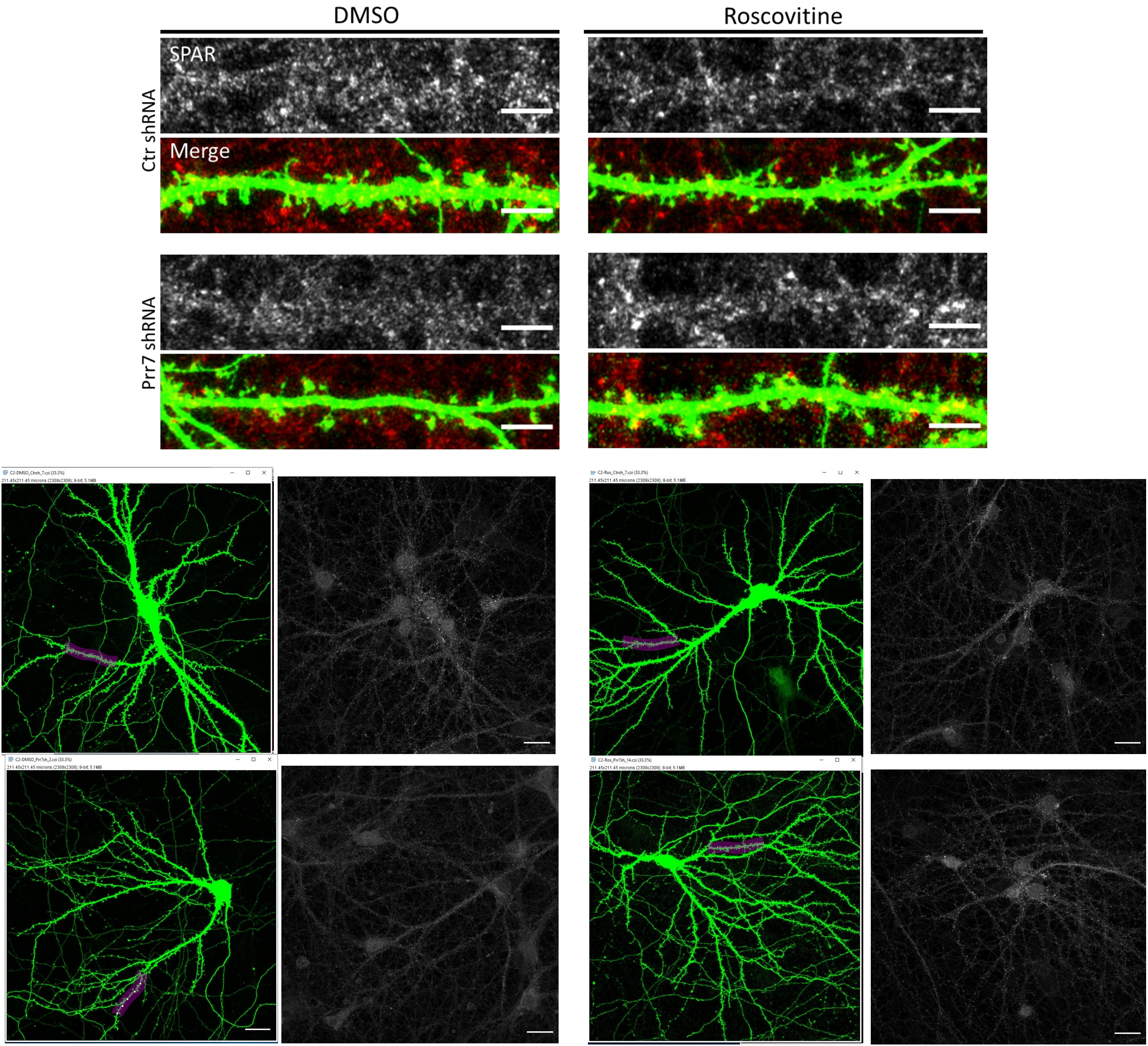
DMSO
Roscovitine
SPAR
Merge
Ctr shRNA
Prr7 shRNA
C2-DMSO_Ctrsh_7.czi (33.3%)
211.45x211.45 microns (2308x2308); 8-bit; 5.1MB
C2-Ros_Ctrsh_7.czi (33.3%)
211.45x211.45 microns (2308x2308); 8-bit; 5.1MB
C2-DMSO_Prr7sh_2.czi (33.3%)
211.45x211.45 microns (2308x2308); 8-bit; 5.1MB
C2-Ros_Prr7sh_14.czi (33.3%)
211.45x211.45 microns (2308x2308); 8-bit; 5.1MB

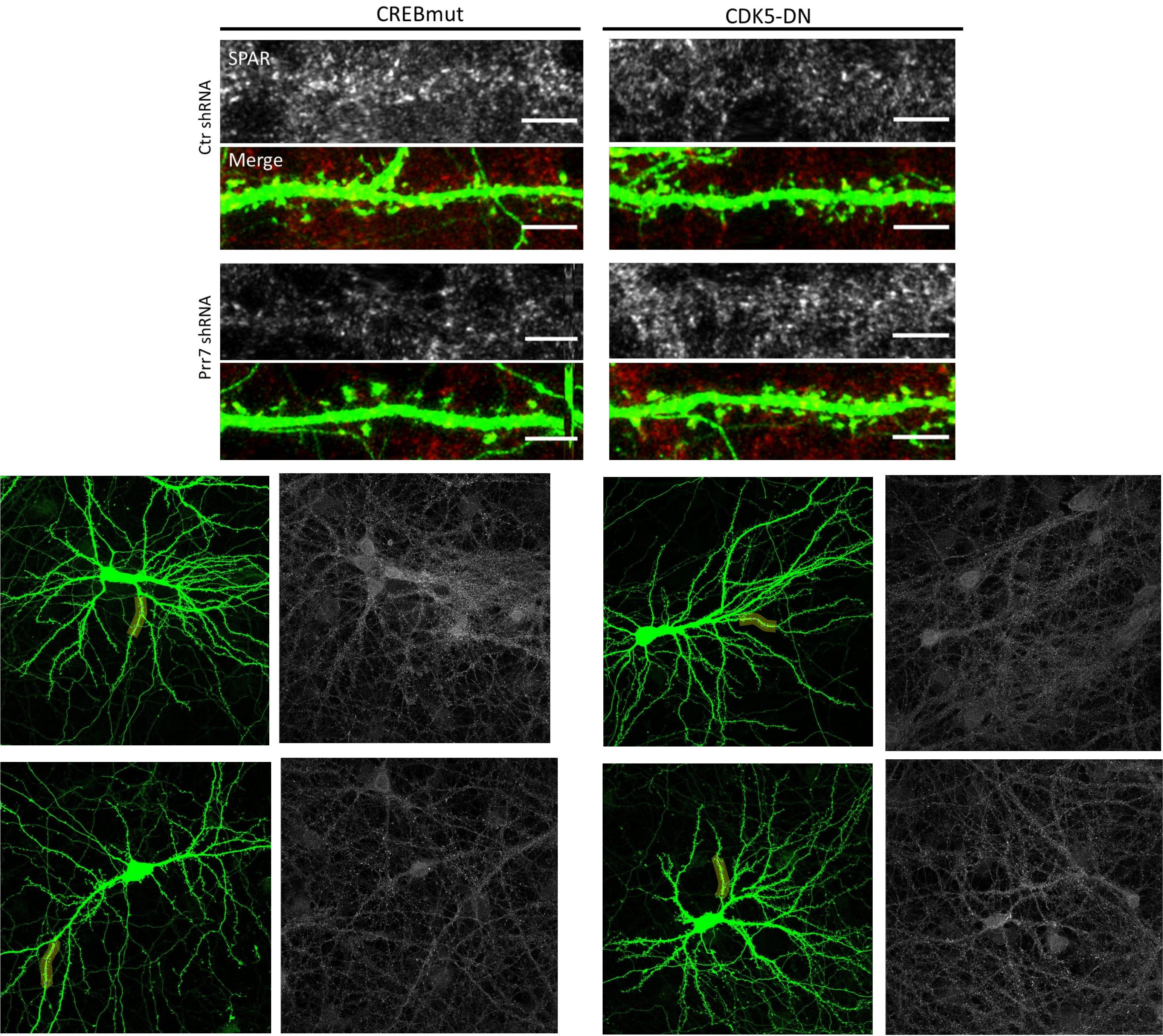

CREBmut
CDK5-DN
Ctr shRNA
SPAR
Merge
Prr7 shRNA
