# Supplementary material for: miR-329– and miR-495–mediated Prr7 down-regulation is required for homeostatic synaptic depression in rat hippocampal neurons
Source: Life Sci Alliance. 2022 Sep 23;5(12):e202201520. doi: 10.26508/lsa.202201520 (PMC9510147; doi:10.26508/lsa.202201520)
Supplement: Supplementary file 13 [file LSA-2022-01520_TableS1.docx]

**Supplementary Table**

**Primer sequences (all 5’ to 3’)**

miR-329 hairpin:

Oligo A-329-BsrGI for: GTACAGCTGTTGACAGTGAGCGACAACACACCC

Oligo A-329-BsrGI rev: AGGTTAGCTGGGTGTGTTGTCGCTCACTGTCAACAGCT

Oligo B-329 for: AGCTAACCTTTTTTGTGAAGCCACAGATGGAAAAAGGT

Oligo B-329 rev: CCCAGAACCTTTTTCCATCTGTGGCTTCACAAAAA

Oligo C-329-HindIII for: TCTGGGTGTGTTGCTGCCTACTGCCTCGGAA

Oligo C-329-HindIII rev: AGCTTTCCGAGGCAGTAGGCAGCAACACA

miR-495 hairpin:

Oligo A-495-BsrGI for: GTACAGCTGTTGACAGTGAGCGACAAACAAACA

Oligo A-495-BsrGI rev: AGTGCACCATGTTTGTTTGTCGCTCACTGTCAACAGCT

Oligo B-495 for: TGGTGCACTTCTTTGTGAAGCCACAGATGGAAGAAGTG

Oligo B-495 rev: ACATGGCACTTCTTCCATCTGTGGCTTCACAAAGA

Oligo C-495-HindIII for: CCATGTTTGTTTGCTGCCTACTGCCTCGGAA

Oligo C-495-HindIII rev: AGCTTTCCGAGGCAGTAGGCAGCAAACAA

Control hairpin:

Oligo A-Ctr-BsrGI for: TGTACAGCTGTTGACAGTGAGCGACAACCTTGTG

Oligo A-Ctr-BsrGI rev: AAGGACCACAAGGTTGTCGCTCACTGTCAACAGC

Oligo B-Ctr for: GTCCTTAGGTGCGTGTGAAGCCACAGATGGCGC

Oligo B-Ctr rev: GGTTTAGGTGCGCCATCTGTGGCTTCACACGCACCT

Oligo C-Ctr-HindIII for: ACCTAAACCACAAGGTTGCTGCCTACTGCCTCGGA

Oligo C-Ctr-HindIII rev: AAGCTTTCCGAGGCAGTAGGCAGCAACCTTG

pSUPER:

Prr7 shRNA targeting rat (and mouse) Prr7 coding region cggaatcggacatgtctaa:

siPrr7_for 1: GATCCCC CGG AAU CGG ACA UGU CUA A TTCAAGAGA

siPrr7_rev 1: AGCTTAAAAA CGG AAU CGG ACA UGU CUA A TCTCTTGAA

siPrr7_for 2: UUA GAC AUG UCC GAU UCC G TTTTTA

siPrr7_rev 2: UUA GAC AUG UCC GAU UCC G GGG

Control shRNA:

siCtr_for1: GATCCCCAAACCTTGTGGTCCTTAGGTTCAAGAGA

siCtr_rev1: AGCTTAAAAAAAACCTTGTGGTCCTTAGGTCTCTTGAA

siCtr_for2: CCTAAGGACCACAAGGTTTTTTTTA

siCtr_rev2: CCTAAGGACCACAAGGTTTGGG

HA-Prr7:

Amplification of rat Prr7 coding sequence with BamHI and XbaI sites:

For: TATAGGATCCGTGATGTCCCAGGGCA

Rev: TCACTCTAGACTATACGGCTGTAGTCCTCCC

Start codon and HA-tag insertion using HindIII site:

For: AGCTT ATG TACCCATACGACGTCCCAGACTACGCT A

Rev: AGCTT AGCGTAGTCTGGGACGTCGTATGGGTA CAT A

shRNA-resistant HA-Prr7:

For: CGG CCC TGG AGC TAT CCG CGC CAA gCC GAG TCA GAT ATG AGT AAg CCG CCG TGC TAC GAG GAG GCG GTG

Rev: CAC CGC CTC CTC GTA GCA CGG CGG cTT ACT CAT ATC TGA CTC GGc TTG GCG CGG ATA GCT CCA GGG CCG

miRNA sensor:

miR-495 mature sequence: AAACAAACAUGGUGCACUUCUU

miR-495 for: GGCCGC aagaagtgcaccatgtttgttt ca aagaagtgcaccatgtttgttt T

miR-495 rev: CTAGA aaacaaacatggtgcacttctt tg aaacaaacatggtgcacttctt GC

(used in Fiore et al., 2009)

miR-329 mature sequence: aacacacccagcuaaccuuuuu

miR-329 for : ggccgc-aaaaaggttagctgggtgtgtt-AC-aaaaaggttagctgggtgtgtt-T

miR-329 rev : ctaga-aacacacccagctaacctttt-GT-aacacacccagctaacctttt-gc

miR-Ctr for : GGCCGCAAGGGATTCTGATGTTGGTCACACTACAAGGGATTCTGATGTTGGTCACACTT

miR-Ctr rev : CTAGAAGTGTGACCAACATCAGAATCCCTTGTAGTGTGACCAACATCAGAATCCCTTGC

(used in Lackinger et al., 2018)

Prr7 3’ UTR luciferase reporter

Prr7 3’ UTR for: AAACTCGAGAGGACTACAGCCGTATAGAGG

Prr7 3’ UTR rev: TTTGTCGACGTACCAAAGCAGATCACACACC

Prr7 3’ UTR mutagenesis was performed for each miRNA binding site sequentially.

Prr7 3’ UTR mut1 for: TACCCTGTTGAATTCATTTTGAGGATAATAAAGG

Prr7 3’ UTR mut1 rev: TCCTCAAAATGAATTCAACAGGGTAAGAAATCC

Prr7 3’ UTR mut2 for: ATAATAAAGGTCTAGAATCTGCTTTGGTACGtCG

Prr7 3’ UTR mut2 rev: ACCAAAGCAGATTCTAGACCTTTATTATCCTCAAAATG

**qPCR primers**

GAPDH for: GCCTTCTCTTGTGACAAAGTGGA

GAPDH rev: CCGTGGGTAGAGTCATACTGGAA

Prr7 for: GTCACGCCCTTTCTGAGC

Prr7 rev: ATGCAGCGCCGAGGTATA

GluA1 for: CGAGTTCTGCTACAAATCCCG

GluA1 rev: TGTCCGTATGGCTTCATTGATG

cFos for: CATCATCTAGGCCCAGTGGC

cFos rev: AGGAACCAGACAGGTCCACATCT
